# Brains at a Premium

**Published:** 1888-02-18

**Authors:** 


### THE DOCTOR'S PULPIT.

### BRAINS AT A PREMIUM.

VERY few men or women of forty fail to understand what is meant when they hear the words "conflict of life," "struggle for existence," "survival of the fittest," and other such expressive and pre-eminently latter-day condensations of human speech. Most of us have found out long before middle age is reached that life is a "stiff fight," and that if we are to have a place among the victorious few we had better give our most potent energies to the business. It is a prime essential to have a plan and a purpose. The writer knows a clergyman who is a man of the highest literary culture and of the finest natural gifts, and yet who is, and always will be, a complete failure because he has neither plan nor purpose in his life-work. He can preach, he can write, he can think, and he can reason with conspicuous ability, but the highest quintessence of brains is entirely lacking. He cannot resolve upon large purposes; he cannot construct and carry out large plans; and so he is in the broadest and profoundest sense, a conspicuous example of failure.

Women are not so capable of reasoning and carrying out formed purposes as men. Nevertheless they have a certain capacity in that direction—a capacity capable of great improvement. What is said hereafter it is hoped may be found as useful for them as for men. Many people think that when they have passed the age of forty it is of no use forming new plans. They imagine they are never likely to have the opportunity of carrying them out, and so they gradually fall into the habit of "letting things drift." That is a mistake of the "foolishest and fatalest" kind, to use a Carlylism. A man should never give up the habit of looking ahead, of forming plans for the future, and of making the most strenuous exertions to get his plans realised. It may be a long time before a particular individual "comes into his kingdom," but if he be a really kingly person, it is surprising how often he actually does come into it—later perhaps rather than sooner—but still early enough to "serve his generation" with some effectiveness.

"Man," it is said, "is a being who looks before and after." A very young man may, and often does, look "before"; it requires a person of some experience to look both "before and after." It is not until a man has acquired the habit of looking both backward and forward intelligently that he answers to this definition of his species. Very few men are competent to form great and wise purposes before they reach middle age. Moses, who carried the Hebrews out of Egypt, and ruled them forty years in the desert, was almost eighty years old at the date of the Exodus. A youthful preacher finds his fittest audience among young people, and an aged one often among kindly and gentle women, but a preacher in the prime of his health and power likes to face the great problems of life in the presence of men of mature age and judgment. They have been compelled to master at least the rudiments of the art of living, and therefore they are prepared to appreciate the more advanced rules and examples.

What is the meaning of "brains" as applied to the affairs of life? By brains the present writer means first of all "eyes"; eyes that see clearly a man's own position, and the possibilities of that position. Here is a lieutenant of twenty commencing his military career. He is a potential Wellington: will he ever be an actual one? That depends upon a thousand circumstances, but two of these outweigh all the other nine hundred and ninety-eight. Has he got capacity and will; or in other words has he got brains? If he has he may one day become a field-marshal in spite of the most stupendous difficulties. If these are lacking he will never be in any true sense of the word a commander of men at all. "Eyes" a man must have to see what it is he can do; and after eyes—"will." It is not strange that so many men fall down and worship an effective will, that even a righteous prophet like Carlyle should pardon all kinds of unrightness in men of great practical capacity. Of course, there is another side to the question; but at present we are concerned with one particular aspect. The utmost possible gentleness and justice are quite compatible with the highest degree of intelligence and the most effective energy of will.

Brains are at a premium always where the population is large and life is lived by rule. This is quite inevitable: a condition of the human-life market which nobody can alter or prevent. It is not desirable that it should be prevented. Intellect, provided it be of the right order, is of the highest positive and relative value, and its natural and healthy development is to be sought by all reasonable means. Every man should seek the improvement of the intellect with which Nature has endowed him. This is the surest way of carrying off, if not the first, at least a worthy prize in life's race. It is a poor spirit which is content to drop into the rear at the very beginning of the contest, or that grows tired before the goal is half reached.

The brains may be improved! How? Not by overwork! They can only be permanently improved by rational training. When a man expects to run a five-mile race he "trains" for months beforehand. Does he begin by rushing off at full speed the very first day, and keeping it up for five miles? If he did the probability is he would never see the end of the training, much less come up to the starting-post on the day of the race. He begins slowly, and increases slowly, never on any occasion doing more than his strength and breathing power will permit. The illustration is simple enough, and its application obvious enough; but how many are there, even among the great medical teachers and practitioners, who practise the principles they all so readily believe and preach? The clergyman who taught his people to do as he said, and not as he did, was no *rara avis*. He was the prototype of hundreds who may be found in every calling, and not least in the medical profession.

The brains are to be improved and developed by reasonable exercise and reasonable rest. The one is as essential as the other. It must be a game of see-saw—up and down, work and rest. What can be more ludicrous and more unsatisfactory to both parties in the game than for one end to be always up in the air and the other always down on the ground? It is just as foolish for a man to overwork his brain as to let it remain altogether unemployed. Moderate work, alternating with moderate rest, gives a brain which, taking the whole life over, will accomplish the most and the best work of which a human being is capable.

Of course in this ill-arranged life of ours the brain-worker, as well as the runner, will sometimes be called upon to make a "spurt." That will do no great harm provided it do not continue too long, and is followed by its appropriate rest or slackening of speed. But here and there people are found who make life all "spurt"; who do no steady running at all. As Napoleon said, "that may be fine, but it is not war"; and so this "spurting" may be magnificent, but it is not life according to the highest rules of art.

Does a man or a woman wish to live well and successfully, doing justice all round and achieving a good record at the last? All creatures endowed with conscience, as we are, should so wish. Whoever aims at a life of duty of this kind must bear in mind that it is not to be done at hap-hazard; it is only possible to reason, prudence, foresight and will—that is to say it is only possible to brains and conscience. The seeing eye, the balancing mind, the potent and unwavering will—these are the generals which lead on to victory; these the highest products of that loom of time which is ever weaving around us the web which constitutes the story of God and of man.

